# gPPIpred: A User-Friendly PPI Predictor Based on Protein Molecular Graphs

**DOI:** 10.17912/micropub.biology.001796

**Published:** 2026-04-10

**Authors:** Cleverson C. Matiolli, Joana Marques, Isabel A. Abreu

**Affiliations:** 1 Instituto de Tecnologia Química e Biológica António Xavier, Universidade Nova de Lisboa (ITQB NOVA), Avenida da República, 2780-157 Oeiras, Portugal

## Abstract

Protein–protein interactions (PPIs) govern essential cellular processes but remain challenging to characterize experimentally due to high cost and labor intensity. We present gPPIpred, a scalable computational framework leveraging graph neural networks (GNNs) and attention mechanisms to predict PPIs at residue-level resolution. Proteins are encoded as spatially informed molecular graphs integrating physicochemical features. Using curated structural datasets for training and validation, gPPIpred was fine-tuned to reliably predict positive interactions and actual interacting sites. Attention scores highlight key residues mediating interactions, offering interpretable insights to guide experimental design. gPPIpred combines high predictive performance with explainability, providing a user-friendly pipeline for large-scale PPI discovery.

**Figure 1. gPPipred: a user friendly, protein-protein interaction predictor:** The gPPipred app, built in gradio, is available at: https://huggingface.co/spaces/1143Joana/gPPIpredv2_2 . **a)** Model Convergence plot showing cross-entropy loss over 20 epochs. **b)** Model Performance measured in MCC over 20 epochs of training. To calculate the MCC during training a balanced test dataset (10,000 samples) was used. MCC – Matthew’s correlation coefficient. **c)** Precision and Sensitivity plot in function of interaction threshold. **d)** Receiver Operation Characteristic Curve. Final AUC-ROC: 0.9109. Optimal Threshold: 0.85. ROC - Receiver Operation Characteristic. AUC - Area Under the ROC Curve. **e)** Confusion Matrix. MCC – Matthew’s correlation coefficient. **f)** gPPipred web app. Users can input proteins by their UniProt Accession IDs. Additionally, users can define their desired threshold. **g)** Here, we show an example of an output generated by gPPIpred. Once the program is finished analysing, the status area indicates how many preys were analysed. The chart shows the probability of each interaction as well as the line for the defined threshold. The interaction is considered positive if probability ≥ than the defined threshold. **h)** Data area. Here, the users can find and download the list of the tested interactors. This list shows bait and prey IDs, the interaction probability, Binds (Yes, if probability ≥ than the defined threshold. No, probability <&nbsp; than the defined threshold), Bait and Prey Hotspots (Hotspot residues are at the interface of either bait or prey proteins that provide the bulk of the binding free energy (ΔG) for the specific interaction pair), and full sequences of Bait and Preys.

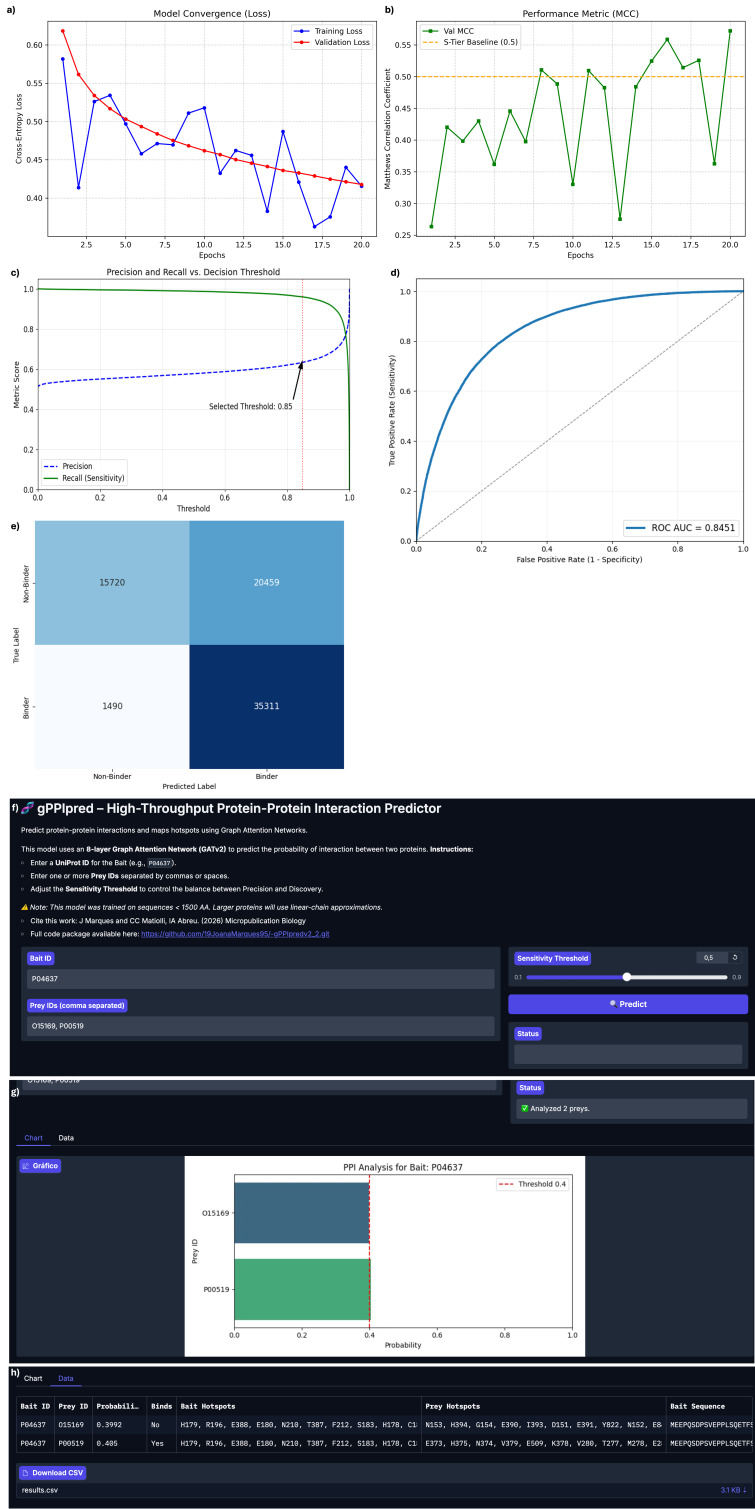



## Description

Protein–protein interactions (PPIs) are fundamental to essential biological processes, including signal transduction, immune response, and metabolic regulation. Traditionally, PPIs have been characterized using low-throughput experimental methods such as Fluorescence Resonance Energy Transfer (FRET), Bimolecular Fluorescence Complementation (BiFC), and yeast two-hybrid (Y2H) systems. High-throughput approaches often use mass spectrometry-coupled techniques, including co-immunoprecipitation (Co-IP) and tandem-affinity purification (TAP) (Bajar et al., 2016; Fields & Song, 1989; Gavin et al., 2002; Ho et al., 2002; Low et al., 2021; Miller et al., 2015). However, these experimental techniques are often labor-intensive, time-consuming, costly, and prone to high rates of false positives and false negatives (Ito et al., 2001; Low et al., 2021; Mrowka et al., 2001). Recently, AlphaFold has provided a crucial advance in protein structural information by greatly increasing the number of high-accuracy protein structure models (Jumper et al., 2021; Varadi et al., 2022). As a result, computational approaches have emerged as efficient, scalable alternatives for PPI prediction.

PPI prediction has relied on scalable computing techniques like machine learning (ML) and deep learning (DL) frameworks. Traditional ML techniques, such as support vector machines (SVM) and Random Forests, have been extensively used for PPI prediction (You et al., 2014, 2015). These methods typically use sequence-based features, including position-specific scoring matrices (PSSM), which capture evolutionary conservation, and physicochemical properties of amino acids to represent proteins (Guo et al., 2008; Shen et al., 2007). While effective, these methods depend on manual feature engineering, which limits scalability and hinders the integration of structural context. Advances in deep learning, particularly convolutional neural networks (CNNs) and recurrent neural networks (RNNs), have improved performance by learning high-level features directly from protein sequences (Hashemifar et al., 2018; Sun et al., 2017). However, these models often underutilize the structural information crucial for capturing the spatial relationships in protein interactions.

Graph Neural Networks (GNNs) are a type of deep learning method that can infer information from graphically represented data. GNNs are now being used to integrate protein structural information with convolutional and recurrent networks to increase prediction robustness (Réau et al., 2021). Both Zhou et al. (2022) and Lee (2023) have summarized and compared methods that use GNN-based strategies (Lee, 2023; Zhou et al., 2022). AlphaFold represents a landmark in the application of GNNs to biological problems (Jumper et al., 2021; Varadi et al., 2022). While AlphaFold was initially used to predict the 3D structure of proteins, it can now be applied to predict complexes between proteins. Its latest update, AlphaFold3, can test interactions between a range of molecules, including DNA, RNA, ligands, ions, and proteins (Abramson et al., 2024). Notably, the model can now account for post-translational modifications and chemical modifications of nucleic acids. All of this is presented in a user-friendly environment, the AlphaFold server, where users only need to input their molecules sequences. However, it does not support high-throughput analysis, as users are limited to 30 jobs per day, and each job cannot exceed 5,000 tokens (1 amino acid =1 token).

Here, we introduce gPPIpred, a novel framework that leverages Graph Neural Networks (GNNs) with integrated attention mechanisms to simultaneously consider physicochemical properties and structural information to predict PPIs. In gPPIpred, proteins are represented as residue-level graphs, where nodes correspond to amino acid residues and edges are established based on spatial proximity within three-dimensional protein structures. Each protein is modeled as a graph, with nodes representing residues of significant structural and functional relevance. Nodes encode the following residue-level physicochemical properties: hydrophobicity, volume, polarizability, pI, and pKa (see Feature Extraction section). The specific values for each amino acid are listed in Extended data Table 1 (Kyte & Doolittle, 1982). Edges in the graph are defined using a spatial threshold. By structuring the graph around both residue properties and spatial threshold rather than individual residues, the model effectively captures biologically relevant interaction motifs. For each predicted interacting pair, interaction hotspots are calculated via saliency mapping (see Methods). Here, the saliency mapping is based on the final interaction probability, providing information on which residues in both prey and bait are critical for that specific interaction.

To iteratively refine residue embeddings, we employed a Graph Attention Network (GATv2) architecture. gPPIpred is a GATv2 model with eight layers of recursive message passing and multi-head attention. Two independent PPI datasets were created to train and validate this model. The breakdown of these datasets is in Table 1. The Siamese neural network was trained for 20 epochs using 128 shuffled graph batches. The use of shuffled batches and independent validation sets ensures the model's ability to generalize across different protein families. Cross-entropy losses observed during training are shown in Figure 1a. During training, the model exhibited a consistent decrease in cross-entropy loss, reaching convergence within 20 epochs.

**Table 1. Number of samples contained in each dataset** . Number of Unique Proteins IDs in each dataset. No protein was used in both datasets to prevent data leakage. The percentage of species represented in each dataset is also listed.

**Table:** 

Dataset	Total&nbsp;	Positive&nbsp; interactions	Negative&nbsp;interactions	Unique Proteins
Training	159,655 interactions: 77.87 % *Homo sapiens* , 22.05 % *Arabidopsis thaliana* , 0.05 % *Oryza sativa* , 0.02 % *Saccharomyces cerevisiae*	77,508 (48%)	82,147 (52%)	10,349 proteins: 64 % *Arabidopsis thaliana,* 35 % *Homo sapiens* , 0.8 % *Oryza sativa* , 0.2 % *Saccharomyces cerevisiae*
					Validation	72,358 interactions:&nbsp; 50.42 % *Homo sapiens* , 49.54&nbsp; % *Saccharomyces cerevisiae*	36,179 (50%)	36,179 (50%)	4,192 proteins: 67.6 % *Homo sapiens* , 32.4 % *Saccharomyces cerevisiae*

The Matthew’s Correlation Coefficient (MCC) values obtained during each epoch are shown in Figure 1b. The GATv2-based Siamese network achieved an MCC of 0.57 during training. To verify the model’s ability to generalize to unseen biological data, we tested gPPIpred on an independent validation dataset of 72,358 protein pairs. The model achieved an MCC of 0.4641, confirming that the Siamese architecture and graph-based representations are not overfitting to specific protein families. The optimal threshold of 0.85 is determined by the point where Precision is maximized without sacrificing the model’s Sensitivity of 96% (Figure 1c). This threshold ensures the model remains a robust screening tool, capturing nearly all true interactions while maintaining an acceptable precision level. The performance metrics – ROC-AUC: 0.8451, MCC: 0.4641, and accuracy: 0.6992—reflect the model’s robust classification ability (Figure 1d). At a 0.85 threshold, performance differed between classes: non-binders (N = 36,179) showed high precision (0.91) but lower recall (0.43; F1 = 0.59), whereas binders (N = 36,801) showed high recall (0.96) with moderate precision (0.63; F1 = 0.76). Overall performance was balanced, with macro and weighted averages of 0.77 (precision), 0.70 (recall), and 0.68 (F1-score). The macro average of 0.70–0.77 across these metrics confirms that the model performs robustly for both classes, despite the distinct challenges each class presents. The high recall (96%) is particularly significant biologically, as it means nearly all true biological interactions are captured. The precision of 0.65 indicates a moderate rate of false positives. The confusion matrix (Figure 1e) shows that the model effectively prioritizes true-positive interactions, making it a viable computational filter for large-scale protein-protein interaction screening.

This level of performance is especially notable given that the training dataset included proteins from diverse species, representing a broad spectrum of the proteome (Table 1). We offer gPPIpred in two ways: as a ready-to-use app accessible here (https://huggingface.co/spaces/1143Joana/gPPIpredv2_2), or by installing gPPIpred (see Extended Data section). To use the app, users only need to provide the UniProt IDs to run a query and define a threshold (Figure 1f). Once the prediction is complete, a bar chart will appear, providing the full list of preys and their interactions probabilities (Figure 1g). Additionally, in the data window, users can find and download the list of tested interactors. This list shows bait and prey IDs, interaction probability, Binds (Yes, if probability ≥ than the defined threshold; No, if probability < than the defined threshold), Bait and Prey Hotspots (hotspot residues are at the interface of either bait or prey proteins and provide the bulk of the binding free energy (ΔG) for the specific interaction pair), and the full sequences of Bait and Preys (Figure 1h).

To install gPPIpred, the following dependencies are required: Python 3.8+, PyTorch, PyTorch Geometric (PyG), and BioPython. We recommend using an NVIDIA GPU with CUDA support due to the high computational demand of graph attention mechanisms; however, execution on a CPU is possible. For the full list of requirements, see the Extended Data section. When using the manually installed version, gPPIpred will generate a link redirecting to an interface like the one shown in Figure 1, making it easy to use once installed.

Despite its strengths, gPPIpred has limitations. For example, it depends on the availability of protein structural data, such as high-quality 3D structures, whether experimental or predicted, which are required to construct accurate graphs. Therefore, gPPIpred should not be used to predict interactions involving proteins that are intrinsically disordered or lack a reliable structural model, as this will yield low-confidence results.

Overall, by leveraging structural representations through graph-based learning and integrating advanced embedding techniques, gPPIpred reduces research costs associated with large-scale interaction screenings and enhances our understanding of the structural determinants of protein interactions. The detailed insights provided by residue-level predictions have important implications for studying the biological mechanisms underlying these interactions, potentially guiding experimental validation and therapeutic targeting. The practical utility of gPPIpred is further enhanced by its interpretability via Saliency Mapping. For the predicted interactions, the model successfully identified residue-level hotspots at the interaction interfaces. The residues assigned the highest saliency scores correspond to those with significant functional relevance, such as those involved in hydrogen bonding or hydrophobic packing at the interface. These "saliency-identified" residues provide a direct roadmap for experimentalists looking to validate predictions through point mutations. Additionally, the gPPIpred pipeline facilitates PPI predictions by offering a simplified interface that automates the download and processing of protein and compound structural files, generates interactive 3D plots to visualize putative interaction sites, and creates detailed reports.

## Methods

**Dataset Preparation**

The training and validation datasets are available in the Extended Data section. Positive interaction data were curated from the Gold Standard Dataset and multi-validated experiments from BioGRID (Bernett, 2022; Chatr-Aryamontri et al., 2015; Oughtred et al., 2021; Szklarczyk et al., 2019). A common strategy for generating negative datasets is Subcellular Localization Filtering, which involves selecting proteins from different subcellular locations and labeling them as non-interacting. Although this strategy is widely accepted, the risk of false negatives can be further reduced by using experimentally validated negative examples. Therefore, we used a negative dataset curated by Russel Lab, dataset Stelzl (2005) (Stelzl et al., 2005; Trabuco et al., 2012). For negative interactions, the shortest path between the two proteins in the underlying two-hybrid interactome is assigned a confidence score in the following format: shortestPath:2, shortestPath:3, etc., or shortestPath:NA if there is no path connecting the two proteins. We created two separate datasets, ensuring that no individual protein sequence appeared in both datasets to prevent data leakage (Table 1). Only interactions where both proteins had high-resolution structures were included. When possible, we ensured the model had examples of both positive and negative interactions for the same protein.

**Protein Graph Construction**

Protein graphs were constructed by representing individual residues as nodes, with edges indicating spatial proximity based on a distance threshold of less than 9.5 Å between alpha carbon atoms. This threshold provides an accurate representation of the protein's tertiary structure and local chemical environment. Preprocessing included extracting Cartesian coordinates (x, y, z) from structural files and standardizing amino acid nomenclature into single-letter codes (Berman et al., 2000). To maximize structural coverage, we integrated experimentally determined structures from the PDB with high-confidence predicted models from AlphaFold2 (Jumper et al., 2021; Varadi et al., 2022). Missing entries were retrieved via the AlphaFold API, resulting in a nearly complete structural dataset. Node features were defined by a five-dimensional vector of physicochemical properties (hydrophobicity, volume, polarizability, pI, and pKa), while edges were defined by Euclidean distances, capturing the spatial constraints essential for predicting protein-protein interactions.

**Feature Extraction**

Node-level embeddings were generated by assigning relative values (0 to 1) for five physicochemical properties to each amino acid (Extended data Table 1). Thus, the model recognizes that 1.0 represents the maximum expression of that specific property. Each physicochemical property provides the model with different information. Hydrophobicity indicates the tendency of an amino acid to repel water, with 1 being the most hydrophobic and 0 the least. Volume is calculated as the Van der Waals volume, where 1 corresponds to the largest and 0 to the smallest amino acid. Polarizability measures how well an amino acid can engage in Van der Waals or London dispersion forces. Mathematically, polarizability (α) is defined as α = p/E, where p is the induced dipole moment and E is the electric field. Colloquially, polarizability reflects how "sticky" an amino acid is. The isoelectric point (pI) is calculated as pI = (pKa1 + pKa2)/2, indicating how basic (1) or acidic (0) an amino acid is. The dissociation constant (pKa) is determined empirically and informs the model whether a residue will be protonated or deprotonated at pH 7.4. These features capture local structural motifs and inter-residue interactions, enabling the model to identify spatial dependencies critical for predicting PPIs. The residue adjacency matrices, constructed with a 9.5 Å spatial threshold, preserved topological structures and emphasized meaningful connections between residues (Kipf & Welling, 2016).

**Graph Neural Network Architecture**

A Graph Attention Network (GATv2) architecture was employed to iteratively refine residue embeddings. Through eight layers of recursive message passing and multi-head attention, the model aggregated neighborhood information into a global graph-level representation (Brody et al., 2021). This process ensured that the final representations were robust descriptors of protein geometry and chemistry, forming the foundation for accurate interaction prediction.

**Training and Validation**

The gPPIpred model was implemented as a Siamese Neural Network, a dual-stream architecture designed to learn relationships between pairs of entities. The gPPIpred GATv2 was trained for 20 epochs in batches of 128 shuffled graphs (Figure 1a and b). Here, we use a Siamese neural network that employs error back-propagation during training; the networks operate in parallel and compare their outputs at the end, usually using cosine distance. The training and validation scripts can be found in the Extended Data section.

**Interaction Site Analysis**

To move beyond "black-box" predictions, we used Saliency Mapping to identify interaction "hotspots." Saliency mapping calculates the gradient of the output probability with respect to the input node features. By visualizing these gradients, we can identify specific residues that contribute most to the predicted binding event. These residues typically correspond to interface regions that provide most of the binding free energy (ΔG), offering actionable targets for site-directed mutagenesis or therapeutic intervention.
